# Treatments for Latrodectism—A Systematic Review on Their Clinical Effectiveness

**DOI:** 10.3390/toxins9040148

**Published:** 2017-04-21

**Authors:** Nicole M. Ryan, Nicholas A. Buckley, Andis Graudins

**Affiliations:** 1 Clinical Toxicology Research Group, School of Medicine and Public Health, The University of Newcastle, Newcastle 2298, Australia; 2 Sydney Medical School, University of Sydney, Sydney 2006, Australia; nicholas.buckley@sydney.edu.au; 3 Department of Medicine, School of Clinical Sciences at Monash Health, Monash University, Melbourne 3800, Australia; Andis.Graudins@monashhealth.org

**Keywords:** red-back spider, widow spider, envenomation, antivenom, treatment

## Abstract

Latrodectism or envenomation by widow-spiders is common and clinically significant worldwide. Alpha-latrotoxin is the mammalian-specific toxin in the venom that results in toxic effects observed in humans. Symptoms may be incapacitating and include severe pain that can persist for days. The management of mild to moderate latrodectism is primarily supportive while severe cases have variously been treated with intravenous calcium, muscle relaxants, widow-spider antivenom and analgesic opioids. The object of this systematic review is to examine the literature on the clinical effectiveness of past and current treatments for latrodectism. MEDLINE, EMBASE and Google Scholar were searched from 1946 to December 2016 to identify clinical studies on the treatment of latrodectism. Studies older than 40 years and not in English were not reviewed. There were only two full-publications and one abstract of placebo-controlled randomised trials on antivenom use for latrodectism. Another two randomised comparative trials compared the route of administration of antivenom for latrodectism. There were fourteen case series (including two abstracts), fourteen case reports and one letter investigating drug treatments for latrodectism with the majority of these also including antivenom for severe latrodectism. Antivenom with opioid analgesia is often the major treatment reported for latrodectism however; recent high quality evidence has cast doubt on the clinical effectiveness of this combination and suggests that other treatments need to be investigated.

## 1. Introduction

Latrodectism or envenomation by widow-spiders (*Latrodectus* spp.), is common worldwide and is the most clinically significant spider envenomation in the United States [1,2,3] and Australia [4,5]. Throughout the world, there are over 40 species of widow-spider, with the red-back spider (*Latrodectus hasselti*) [6] in Australia and the black widow spider (various *Latrodectus* spp.) in the United States of America [7] being of most clinical concern. However, species are found on all continents except Antarctica. The venom contains alpha-latrotoxin (α-LTX), a mammalian-specific neurotoxin that binds to presynaptic nerve terminals and stimulates massive neurotransmitter release Two classes of receptors for α-latrotoxin have been identified. Neurexins are brain-specific proteins that partly function as cell adhesion molecules binding α-latrotoxin in a Ca^2+^ dependent manner. CLs (CIRLs and latrophilins) are not brain-specific but widely expressed in all tissues and bind α-latrotoxin independently of Ca^2+^ [8,9]. Khvotchev and Südhof recently showed that α-latrotoxin inserts into the presynaptic plasma membrane after receptor binding, resulting in an intracellular location of the N-terminal sequences triggering neurotransmitter release [10]. Alpha-latrotoxin is most likely responsible for the majority of the clinical effects of latrodectism. Envenomation by the *Latrodectus* species can result in an incapacitating syndrome of severe local, regional or systemic pain and autonomic features that, if left untreated, may last for several days. Severe and persistent pain occurs in a half to two-thirds of cases of latrodectism [11] and is the primary target of treatments. Treatment has ranged from intravenous injection of calcium gluconate to muscle relaxants such as benzodiazepines with opioid analgesia to specific treatment with antivenom, such as red-back spider antivenom (RBSAV, purified IgG-F(ab)_2_) and black widow spider antivenom (whole IgG *L. mactans*). There continues to be concern regarding the risk of acute severe allergic reactions and serum sickness with antivenom use, and adverse drug effects with the use of benzodiazepines and opioids. The aim of this review is to examine the literature on the effectiveness of clinical treatments for latrodectism.

## 2. Clinical Characteristics of Latrodectism

Symptoms of latrodectism are mediated by the neurotoxin alpha-latrotoxin. Latrotoxin causes neurotransmitter release through both calcium-dependent (Neurexins) and calcium-independent (latrophilin) mechanisms [8,9]. This results in skeletal muscle and autonomic effects. However, the most troublesome and predominant feature of systemic latrodectism is local and regional pain, which classically increases over hours. In Australia, local pain radiating up the bitten limb or from the bite site is typical, whereas in North and South America back and abdominal pain predominate [5].The pain is accompanied with nonspecific systemic effects such as nausea, vomiting, headache, malaise and lethargy, local and regional diaphoresis, and less commonly other autonomic neurological effects [5,11]. Diaphoresis often occurs in unusual patterns that are almost pathognomonic for latrodectism—Diaphoresis localised to the bite site, bilateral below-knee diaphoresis, and asymmetrical regional diaphoresis are some characteristic patterns. Nonspecific systemic effects occur in approximately one-third of the cases. Hypertension, agitation, fever, priapism, patchy paralysis, paraesthesia, muscle fasciculations, and cardiac effects less common [11].While the severity and some features of latrodectism vary for different widow spiders from different regions, [1,4,11,12] pain is the most prominent feature in all cases. Early control of pain is paramount as nociception elicits important physiological responses and reduction of pain can improve clinical outcomes and prevent chronic pain [13].

## 3. Earlier Treatments for Latrodectism

Throughout most of the 20th century (particularly in North America) calcium was considered a first-line treatment for latrodectism even though there have been no placebo-controlled clinical trials to demonstrate its effectiveness [14].Indeed, calcium was once proposed as an “antidote” for alpha-latrotoxin by virtue of its proposed efficacy in vitro [15].However, subsequent clinical experience has suggested that calcium may not have the same effect clinically, resulting in a loss of confidence in its effectiveness [1].

Five case series and one case report were identified investigating the treatment of latrodectism with drug treatments such as calcium gluconate, muscle relaxants and opioid analgesia in the past 40 years. These are summarized in (Table 1), with study design problems identified. A more detailed summation on some of these studies follows.

The earliest study reviewed was a case series by Key in 1981 [16]. Calcium gluconate and methocarbamol, a muscle relaxant, were compared using an algorithmic treatment protocol on all cases of *Latrodectus* (black-widow) envenomation seen over a period of three years. According to the treatment algorithms, patients received calcium gluconate alone or after methocarbamol failure. Six of 13 patients treated according to this algorithm were assessed as effectively treated by the attending physician. Ten patients were treated with methocarbamol alone or after calcium gluconate failure according to the second protocol. One of these patients was assessed as effectively treated. Key concluded that calcium gluconate should remain the treatment of first-choice.

Ryan (1984) [17] reported on eight patients with severe muscular pain after bites by black widow-spiders that were treated with the muscle relaxant dantrolene sodium. Six patients received the medication both intravenously and orally while two patients received the oral preparation only. Intravenous doses in this study varied between 0.5 mg/kg and 0.9 mg/kg and oral doses varied between 25 mg and 100 mg given every four hours for 2–4 doses. Five of the six patients given both intravenous and oral dantrolene sodium had good muscle relaxation. The two patients given only oral doses had less satisfactory results. Overall it was noted that symptoms were more pronounced and more protracted than had generally been reported with centrally acting muscle relaxant treatment.

Timms and Gibbons (1986) [18] reviewed the medical records of 11 patients admitted with a diagnosis of latrodectism. Patients ranged from 14 to 60 years and recovery time was defined as the interval between treatment and relief of symptoms. Positive identification of the spider *L. mactans* was either made on a specimen by the ED physician or by an accurate description of the spider by the patient. All patients had muscular pain; abdominal pain was experienced by 7/11 patients. Nausea and vomiting occurred in four, headache in three and dyspnea in two. Most patients were seen within two-hours. Nine of 11 patients received 10–20 mL of calcium gluconate in a 10% solution intravenously in addition to muscle relaxants (diazepam or methocarbamol), one received methocarbamol, meperidine hydrochloride and nasogastric suction and one received calcium gluconate and methylprednisolone sodium succinate.

Two of the 11 patients also received *L. mactans* antivenom intravenously in addition to the calcium gluconate and muscle relaxant. Of the 11 patients, seven required narcotic analgesics for relief of pain. The authors concluded that latrodectism is generally self-limiting and responds to calcium and muscle relaxant administration. While compromised patients can be treated with antivenom, it may result in serum sickness.

Recommendations in a 1989 review by Binder [19] further advised the treatment of ‘common’ envenomation with 10% calcium gluconate intravenously titrated to relief of symptoms. Due to the significant chance of hypersensitivity and serum sickness reactions with North American widow-spider antivenom it was advised that this should be restricted to life-threatening cases only. The frequent use of calcium therapy for latrodectism was questioned a few years later largely based on the case series by Clark et al. (1992) [1], (Table 2). Their report was a notably a more comprehensive evaluation compared with earlier studies. Firstly there was a much larger number of cases of black-widow spider envenomation investigated, *N* = 163. Patients were categorized on severity depending on their clinical presentation. This included symptom severity, physical findings and vital signs. A positive identification of the spider was recorded in most (72%) cases. Measurable outcomes such as the average time from envenomation until onset of symptoms (1.2 ± 1.6 h) and the average time from onset of symptoms to hospital presentation (6.2 ± 7.8 h) were recorded. The most common complaint on presentation was generalized muscular abdominal, back and leg pain and 36 patients were diaphoretic. Forty-five patients were hypertensive with only six of these having a history of hypertension. Fourteen patients presented with tachycardia. Twenty-two patients were sent home after evaluation in the hospital Emergency Department (ED) but returned to an ED with recurring pain. Pain relief was recorded as being most effective with either black widow spider-specific antivenom (*L. mactans* AV) either alone or in combination with IV opioids (IV or IM morphine and/or meperidine) and muscle relaxants (benzodiazepines). A total of 58 patients received antivenom with complete resolution of symptoms in a mean time of 31 min from the end of the infusion, this was significantly shorter than patients not treated with AV. Fifty patients reported pain relief after one vial and required no further pain medication whereas seven patients required an additional vial. No patient needed more than two vials of *L. mactans* AV. One patient died of severe bronchospasm after receiving antivenom and had a history of asthma. Calcium gluconate was found to be ineffective with most moderate to severe envenomation cases requiring the addition of IV opioids or other analgesics for symptomatic relief. This finding was supported through later case reports, including one by O’Malley et al. in 1999 [20], that detailed the treatment of latrodectism in a 13 year old male bitten by a black-widow spider while sleeping. The patient was initially treated in the ED of a nearby hospital with calcium gluconate and analgesia with minor pain relief resulting. Although the patient was unable to walk he was discharged only to present three days later at another ED where he was treated with one vial of *L. mactans* IV antivenom 1 L of normal saline infused at a separate site. The authors report a successful response to the antivenom 10 min later. It was concluded that administration of antivenom to patients with prolonged or refractory symptoms of latrodectism may alleviate discomfort and weakness.

As a result of these reports calcium treatment of latrodectism declined and gave way to alternative treatments that appeared to be much more effective for symptomatic relief. Opioid therapy in combination with muscle relaxants for patients not eligible for antivenom treatment became the recommended treatment for latrodectism [21,22] while antivenom was primarily reserved for severe envenomation due to concerns of severe allergic reactions to whole IgG molecule Antivenom.

## 4. Antivenoms and Their Mechanism of Action

Antivenoms are antibody preparations that are produced from the plasma of animals, usually horses or sheep, by injecting the animals with venoms. Due to their polyclonal nature antivenom is able to neutralize multiple toxins within the venom [23]. Antivenoms can be either whole IgG molecules, F(ab’)_2_ fragments or Fab fragments. Proposed mechanisms of how antivenom works in humans includes blocking the active site of a toxin or binding to a toxin to prevent it interacting with its substrate thereby neutralising the toxin. Central vascular compartment antivenom-venom complex formation may prevent distribution of toxins to the target tissues such as the nervous system or cause redistribution of the toxins from target tissues back to the vascular compartment. Antivenom may also increase the rate of toxin elimination from the body depending on the relative clearance of the antivenom and toxin [23,24].

A number of antivenoms produced against the venoms of specific *Latrodectus* spp. have been developed in various regions around the world. These include the black-widow spider (*L. mactans*) antivenom of North America (Merck), the red-back spider (RBS, *L. hasselti* antivenom) in Australia (CSL, Ltd., Melbourne, Australia), the black-widow (*L. indistinctus*) and brown-widow (*L. geometricus*) spiders of South Africa (SAFR), the Argentinian *L. mactans*, and the Mexican widow spider (Aracmyn polyvalent antivenom for latrodectism and loxoscelism), and the discontinued Yugoslavian widow-spider AV (*L. tredecimguttatus*) [25]. The ‘efficacy’ of antivenom is defined as its ability to bind and neutralise venom-mediated effects under ideal conditions (in vitro studies and animal studies of binding and neutralisation), while the ‘effectiveness’ of antivenom is defined as its ability to reverse or prevent envenoming in human patients [26]. There is insufficient prospective randomised controlled study evidence to lend support to the effectiveness of widow spider antivenoms. However, a long history of relatively safe use and anecdotal or retrospective observational study evidence means that antivenom continues to be used for severe cases of latrodectism. More recent randomised controlled trials and a prospective case series of widow spider antivenoms support the safety of the antivenom. These trials suggest that acute allergic-type reactions occur in about 5% of cases, including anaphylaxis in 1–2% and delayed reactions or serum sickness in up to 10% of cases [4,27,28,29]. The efficacy of widow-spider antivenom has been confirmed in the laboratory by Graudins et al. [30] for red-back spider antivenom and for *L. hesperus* and *L. mactans* venoms in a mouse envenomation model by Daly et al. [31].

### 4.1. Black Widow Spider Antivenoms

The first available black-widow spider antivenom was produced by Mulford Biological Laboratories of Sharp and Dohme in the United States in 1936. This product was a lyophilized normal horse serum. After a merger with Merck in 1953, an equine derived whole IgG *Latrodectus mactans* antivenom was produced [14]. *L mactans* antivenom has been used to treat envenomations from various widow spiders including *L. hesperus*, *L. variolus*, and *L. bishop*. The preferred route of administration of *Latrodectus* spp. antivenoms varies from region to region. American black-widow antivenom is administered intravenously and has been associated with adverse effects more frequently than antivenom made elsewhere. One report suggests that the anaphylaxis risk is 9% in those skin testing negative and 80% in those skin testing positively, with a serum sickness rate of 36% [19]. A more recent review [15] suggests that the black-widow antivenom has a similar safety profile to red-back spider antivenom. In a 2012 review by Monte [14] it was revealed that the previously quoted incidence of adverse reactions with the Merck black-widow spider antivenom were actually extrapolations from studies on the whole IgG Wyeth Crotalidae antivenom. Further, it is expected that a purified F(ab)_2_ *L. mactans* antivenom called Analatro^®^, will have fewer adverse reactions when compared to the Merck IgG product. A lack of allergic outcomes observed in a phase II clinical trial of Analatro^®^ in 24 patients by Stanford et al. [28] supports this. Despite this only 3.8% of patients with clinical effects attributed to Latrodectism received antivenom from 2000 to 2008 [7] primarily due to the fear of adverse reactions to the antivenom [1,15,25,32]. There have been two recent reports of anaphylaxis to *L. mactans* antivenom (Antivenin^®^; Merck and Co., West Point, PA, USA) for black widow spider envenomation [33,34]. 

### 4.2. Red-Back Spider Antivenom

In Australia, red-back spider antivenom (RBSAV) has been available for use in the treatment of latrodectism since 1956 [35]. RBSAV is an equine-derived antibody composed of purified IgG-F(ab)_2_ fragments produced by Commonwealth Serum Laboratories (CSL Ltd., Melbourne, VIC, Australia). RBSAV neutralizes 1 µg of *L. hasselti* venom per unit of antivenom in vitro. It has been reported to reverse envenomation by other widow spiders including the European widow spider *L. tredecimguttatus* [30] however, aside from a much earlier report by Keegan [36] the efficacy of non-species-specific antivenom for the treatment of widow spider envenomation was relatively unknown.

Graudins et al. (2001) assessed the in vitro and in vivo efficacy of RBSAV in the prevention of toxicity resulting from other widow spider venoms [30]. In this study, the binding of RBSAV to five *Latrodectus* spp. venoms and α-latrotoxin was assayed using Western blotting. Prevention of in vitro and in vivo toxicity were tested in isolated chick biventer cervicis nerve-muscle preparation and male mice, respectively. In Western blots, RBSAV bound to purified α-latrotoxin and similar molecular weight widow spider proteins in all venoms tested indicating antigenic similarity with proteins found in RBS venom. The antivenom also prevented typical in vitro muscle contracture and loss of twitch tension seen with α-latrotoxin and the all venoms tested. Compared to control envenomed mice, mice inoculated with venom pre-mixed with RBSAV remained free of envenomation signs. From this data it was suggested that RBSAV may be clinically effective in the treatment of envenomation from other widow spiders [30].

## 5. Clinical Studies of Antivenom Effectiveness

### 5.1. Non-Randomised Clinical Trials

Most of the case series (10 from 14), and case reports (9 from 14) discuss the effectiveness of antivenom treatment for latrodectism. This includes three paediatric case reports and two paediatric case series and one letter. The larger of these studies are summarized in Table 2.

The largest case series of antivenom treatment for latrodectism was in Australia by Sutherland and Trinca (1978). They reported on 2144 case reports made to the red-back spider antivenom (RBSAV) manufacturer Commonwealth Serum Laboratories (CSL)™. Over eight years, an average of 240 cases per annum were reported. Local pain, redness and swelling were the most common symptoms. General effects included nausea, vomiting and sweating. Coma and respiratory failure were very uncommon. The investigators attributed this to the use of specific antivenom within 24 h of the bite in 92% of cases and within 2 h in 70% of cases. Only forty-four patients received antivenom intravenously. In the majority of cases, (2073) the intramuscular route was used [37]. There were 11 cases (0.5%) with anaphylactic reactions reported, with no deaths from venom or antivenom reactions. A similar number of delayed antivenom reactions were reported. This study was based on cases treated with antivenom and then reported to the antivenom manufacturer. As a result, patients with greater initial severity and a favorable treatment response may have been reported. Jelinek et al. (1989) [38] conducted a retrospective study of one hundred and fifty patients admitted to hospital with a definite red-back spider bite in Western Australia. Thirty-two (21%) patients received antivenom with 11 (34%) receiving more than one ampoule. This was in contrast to the Sutherland and Trinca series where only 3% of patients received more than one ampoule. The authors stated that this result suggested that the Western Australian red-back spider was more venomous compared to other Australian red-back spiders as more antivenom was needed for a clinical response. The alternative interpretation, that the effectiveness of the antivenom was less than originally believed, was not considered. Indeed it is difficult to understand how RBSAV effectiveness was reported in these two studies as, criteria and time course for ‘treatment success’ were only vaguely specified. RBSAV effectiveness in the Jelinek et al. study was defined as patients becoming ‘asymptomatic’ ‘sometime’ after antivenom administration [39]. In the Sutherland and Trinca study favourable comments such as ‘good’, ‘improved’, ‘successful’ ‘cured symptoms’ written on the AV pack questionnaire were considered evidence of a treatment success. It is interesting to note that 1185 cases were deemed successful in the Sutherland and Trinca report, but there was no improvement reported in 68 cases and no comment on efficacy reported in a further 809 cases from a total of 2062 patients that definitely received (pack questionnaire completed) RBSAV [37].

In the United States, Nordt et al. (2010) [48] performed a retrospective review of a poison centre electronic database from January 1999 to December 2009. All cases of black-widow spider envenomation treated with *L. mactans* antivenom (Merck) were included. Age, gender, signs and symptoms, adjunctive therapy, number of vials of AV given, response to AV, and adverse reaction to AV were recorded. The aim of this study was to determine rate of adverse effects and the efficacy of AV in patients treated for latrodectism, as AV use had been previously limited due to concerns of possible severe allergic reaction. Ninety-six adult and paediatric patients were treated with *L. mactans* AV. No patient required more than one vial of AV. One patient developed urticaria to the antivenom halfway through the infusion and this was immediately discontinued. Another patient developed generalized erythema following completion of infusion but no other effects. There were no deaths in any patients receiving AV. All patients reported pain relief with AV and did not require additional AV doses. Adjunctive therapies included opioids 69%, benzodiazepines 64%, calcium 21%, NSAIDs 17%, and other muscle relaxants 11%. No cases of serum sickness were reported. From this study the investigators concluded that treatment for black widow spider envenomation includes opioid pain control and muscle relaxants and while these medications can provide symptomatic relief they do not neutralize the venom. It was advised that definitive treatment include antivenom although they noted that adequate pain control was often difficult to achieve. Hypersensitivity reactions appeared to be mild and uncommon, however the authors recommended that further prospective studies were required to confirm these results.

In a more recent retrospective review Basanou et al. (2015) [49] investigated *L. mactans* exposures reported to the National Poison Centre of Greece. Fifty-three patients ranging from 2–74 years were reviewed. Symptoms typically lasted for 1–3 days and included abdominal pain (52%), muscle rigidity/cramping (43%), diaphoresis (43%), and other symptoms attributable to the envenomation. Moderate to severe symptoms were treated with IV benzodiazepines (*n* = 6), IV opioids (*n* = 5) or combination of IV opioids with benzodiazepines (*n* = 12). *L mactans* antivenom was administered in five patients with severe systemic symptoms. Treatment relieved pain in 48% of patients taking opioids or benzodiazepines alone, and in 52% using opioids with benzodiazepines. All five patients receiving AV reported complete symptom resolution after 80 ± 30 min. The investigators concluded that opioid analgesics combined with muscle relaxants (benzodiazepines), are generally effective at symptomatic control and in selected severe cases antivenom was the most efficacious treatment available.

Mead & Jelinek (1993) [39] aimed to describe the pattern of illness caused by red-back spider bites to children in Perth, Western Australia over a 10 year period and to compare it with that in adults. The case-notes of 241 children with suspected RBS bite were reviewed. A strength of this study was the defining of a ‘definite RBS bite’ according to the following: (1) definite bite by a positively identified red-back spider; or (2) positive identification of a red-back spider with no definite bite but the later development of typical symptoms; or (3) no definite history of red-back spider bite but strong clinical evidence and complete recovery after administration of antivenom. Sixty-five percent of children were definitely bitten. The syndrome produced in children was usually similar to that seen in adults. Twenty-one percent of children received antivenom, a rate comparable to previous studies in older age groups [38]; however, no child received more than one ampoule and it was deemed that no child remained unwell after a single dose of antivenom. These findings suggest that, contrary to current opinion at the time [40,41,42,43], children may not be at an increased risk of morbidity from latrodectism. The limitations of this study largely reflect the problems of retrospective case reviews, with a lack of uniform recording of signs/symptoms and response. Importantly there was no quantification of antivenom effectiveness. Of those children not treated with antivenom (209/241), most (67%) were observed and given no treatment.

Trethewy et al. (2003) [46] reviewed the case notes of children under 12 years of age with a discharge diagnosis of red-back spider bite over a 8½ year period from a central Australian hospital. There were 54 patients, 39 Aboriginal and 15 non-Aboriginal. Cases enrolled in the study were stratified on the basis of history of bite. A definite bite was recorded if the carer positively identified an RBS and observed it bite the child, or a positive identification of the spider with no definite bite, but the later development of typical symptoms of latrodectism, necessitating review. No definite history of RBS bite was recorded if there was no historical evidence of bite, but strong clinical evidence for envenomation, with complete resolution of symptoms after administration of specific antivenom. Results were stratified to allow comparison of envenomation profiles for different ages and average body mass. Symptoms and signs were also stratified into local and systemic features of envenomation consistent with previous studies [37,39]. Forty-six children (85%) had systemic envenomation. The three most common systemic features were irritability, hypertension and sweating and 35 children (65%) had all three symptoms. There was no significant difference in the clinical characteristics and outcomes of envenomation between the Aboriginal and non-Aboriginal children. Forty-five (83%) children received antivenom therapy (RBSAV), 39 (72%) received one ampoule, and six (11%) required two ampoules. To protect against AV acute adverse reactions, twenty-five (56%) children received an antihistamine premedication 15–30 min before administration of antivenom while sixteen children (36%) received a combination of antihistamine, and adrenaline. Four children (9%) received no premedication. In all cases the antivenom was reported to be efficacious, with complete resolution of the signs and symptoms of envenomation. There were no reports of hypersensitivity reaction in any child treated. This study provided a comprehensive description of the clinical features and outcomes of RBS envenomation in children but did not quantify the time course of the outcome nor have a comparator treatment group.

According to the product information, RBSAV is to be administered undiluted intramuscularly. More severe cases of envenoming are commonly treated with diluted antivenom intravenously. However, some cases have received undiluted antivenom intravenously. While RBSAV has a very low acute reaction rate, half of the allergic reactions reported by Sutherland and Trinca (1980) following its use were attributed to administration of undiluted antivenom by the intravenous route [30,37]. Case studies have reported multiple ampoule use of RBSAV to treat severe envenomation without significant adverse reactions [37,38,50]. Isbister (2007) [29] reviewed the safety of appropriately given dilute IV RBSAV in a larger study of ninety-five patients including 13 children. All patients had local pain, 68 had radiating pain, 54 had diaphoresis and 37 had systemic effects from the latrodectism. Prior to the IV antivenom, forty-two patients had received at least one vial of RBSAV intramuscularly. The median dose of IV RBSAV was two vials. Four of the patients had immediate systemic hypersensitivity reactions after commencement of the IV RBSAV. No patient had a severe reaction, one had a moderate reaction and three had mild reaction. Three of 32 patients followed-up 1–2 weeks later developed symptoms of serum sickness [29]. Antivenom concentrations on serial blood samples using an enzyme immunoassay following IV and IM administration of RBSAV in envenomed patients was also tested by Isbister et al. (2008) [51]. In the serum samples of 10 patients who had received IM RBSAV, immunoassay did not detect antivenom at any time point after one or more vials had been administered. In ten patients who received one or more vials of IV RBSAV the antivenom was detected in all patient serum samples. Consistent with earlier human and animal studies of intramuscularly administered antivenom, it appeared that IM RBSAV did not reach the systemic circulation and as a result may not be effective [51].

### 5.2. Randomised Clinical Trials of Antivenom for Latrodectism

We identified three placebo-controlled randomised trials [52,53,54] and two no-placebo randomised comparator trials [4,27] of antivenom for Latrodectism (Table 3).

**Figure 1:**
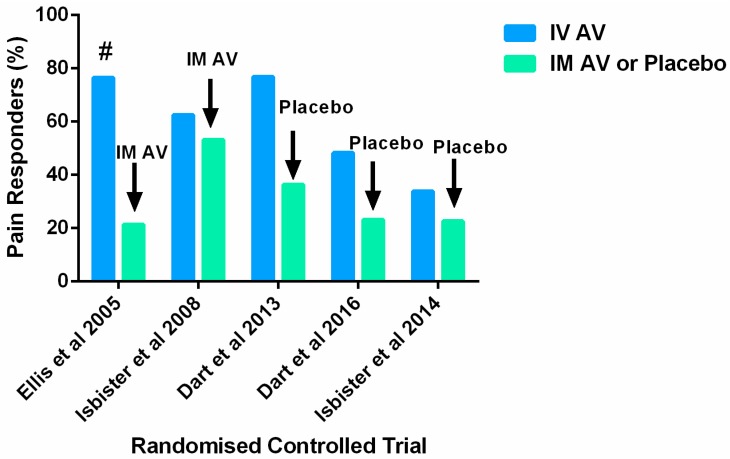
Graph of Pain Responders (Treatment Success) to IV Antivenom compared to Pain Responders (Treatment Success) to IM Antivenom or Placebo. # response at 24 h not at specified primary outcome.

In the first full-publication no-placebo randomised comparator trial, Ellis et al. (2005) [27] randomised eligible patients across five participating sites to RBSAV by IM injection (*n* = 15) or RBSAV by IV infusion (*n* = 18). The aim of this study was to determine which route of AV administration was more effective for treating latrodectism. This study was very under-powered, two patients dropped out and more pre-antivenom analgesia was used in the IV group (8/17, 47%) resulting in lower baseline pain scores than for the IM group where (3/14, 21%) had pre-AV analgesia. The primary 1-h pain measure outcome was actually a 30-min pain measure for the IV group given the time taken to run the infusion. Six from 14 patients in the IM group and one from 17 patients in the IV group did not have pain scores recorded at 2 h also limiting the value of comparison at this point. Previous studies claimed that the majority of patients with RBS bite (between 66% and 97%) were adequately treated with one ampoule of antivenom. Using a protocol that repeated treatment in non-responders, this trial found only a small proportion of patients (21%) responded to only one ampoule. Twelve from 39 patients who remained in the trial after randomisation required IV rescue therapy making the endpoint number of ampoules administered much less useful. The mean number of antivenom ampoules used by the two groups was similar (IM = 2.5 ampoules; IV = 2.6 ampoules), the maximum number of ampoules used was seven. Irrespective of the methodological problems mean pain scores on a 100 mm visual analogue scale were seen to fall over the first hour after treatment for both groups (IM VAS: 67 mm pre-AV to 34 mm post-AV) versus (IV VAS: 54 mm pre-AV to 41 mm post-AV) with no significant difference between the two groups found. At 24 h significantly more patients in the IV group (13/17; 76%) were pain free than in the IM group (3/14; 21%), Figure 1. Length of stay was less than one day for all patients although three IM patients re-presented to hospital over subsequent days for more antivenom. Minor adverse effects were relatively common, including itch, nausea and shivering and one patient in the IV group had flu-like back pain for 10 days (serum-sickness reaction). No adverse event required treatment. The investigators were unable to conclude which route of administration for antivenom was better. The authors suggested that both routes were equally effective at relieving initial pain. However, this may also be interpreted that both routes were equally ineffective, given the overall need for repeated treatments.

The ‘RAVE’ study by Isbister et al. (2008) [4] also compared IM and IV antivenom administration for RBS bite in a considerably larger clinical trial than that of Ellis. This multicentre, randomised double-dummy, double-blind study had a primary outcome of clinically significant reduction in pain 2 h after treatment. All patients received the same pre-antivenom analgesic regimen, the method of administration was randomised and repeated clinical assessments were performed at 30 min intervals after completion of treatment. Sixty-two patients received IM antivenom and 64 received IV antivenom. After antivenom treatment, pain improved in 40/64 (62%) in the IV group versus 33/62 (53%) in the IM group, (Figure 1). In 55 patients with systemic effects, pain improved in 58% after IV antivenom versus 65% after IM antivenom. Twenty-four hours after antivenom, pain had improved in 84% of the IV group and 71% of the IM group. A meta-analysis, including data from the Ellis et al. study, found no difference in the primary outcome between IV antivenom (probability of success 55%) and IM antivenom (probability of success 51%) [4].

Dart et al. (2013) [53] conducted a multi-centre randomised, placebo-controlled clinical trial in patients at least 10 years old with moderate to severe latrodectism following black-widow spider bite. Subjects had to present for treatment within 72-h of symptom onset with a clinical diagnosis of latrodectism made by an investigator and an independent physician. Subjects also needed to have a visual analogue scale (VAS) pain score of at least 40 mm on presentation screening and immediately before infusion of the study drugs. From 24 subjects, 13 were randomised to a single 10-min IV infusion of three vials of antivenom and 11 to placebo IV infusion. This was a phase-2 clinical trial of a F(ab)2 antivenom (Analatro) not yet available for clinical use. Pain was assessed using a 100 mm VAS. The primary outcome was a median change in VAS at 150 min post-treatment. This was −50 mm in the antivenom group and −46 mm in the placebo group (no statistically significant difference). A secondary outcome was time to a clinically important decrease in pain defined as a decrease of at least 13 mm in posttreatment VAS pain score compared with baseline VAS pain score. The median time to a clinically important decrease in pain after treatment was significantly shorter in the antivenom group (30 min [IQR 30, 60 min]) compared with the placebo group (90 min [IQR 30, 90 min]) (*p* = 0.03). A second measure of analgesic efficacy was a comparison of the proportion of patients who did not reach a threshold of pain relief by end of the study period (‘treatment failures’). In this study the proportion of treatment failures in the placebo group was 64% compared with 23% in the antivenom group, (Figure 1). No serious adverse events or deaths were reported for either intervention and there were no significant differences in occurrence or relatedness of adverse events within the two treatment groups. Design limitations of this study included a small sample size, the average time difference between presentation and treatment for the patient groups—patients in the antivenom group presented on average 4-h later than the placebo group. The investigators acknowledge that this may have reflected a difference in disease severity or could have altered the treatment efficacy or natural progression of disease during the study period. A further study limitation was protocol non-compliance. There were 23 protocol deviations reported in 11 subjects (five in the AV group and six in the placebo group). These deviations included incorrect administration doses of allowable analgesics and consequently, inconsistencies with the recording of the pain score. The investigators suggest that these violations, whilst prevalent in both groups, may have biased the study toward finding no difference between the study groups. Finally the difficulty in diagnosing latrodectism was mentioned as a limitation of this study. This study was partly funded (and one of the authors employed) by the manufacturers of Analatro F(ab)2 antivenom [Instituto Bioclon S.A. de C.V., Talapalan, Mexico].

The most recent full-publication multi-centre randomised placebo-controlled clinical study was performed by Isbister et al. (2014) [54]. This study investigated the effectiveness of red-back spider antivenom for latrodectism in 224 patients over 7-years of age. Inclusion criteria were specified: a red-back spider bite where the treating clinician would normally administer antivenom or analgesia for the pain, or for systemic envenoming. Red-back spider bite was pre-defined as either a bite by a spider that was clearly identified as a red-back spider (by the patient or clinician) or a clinical syndrome consistent with typical red-back spider envenoming, that is, the sensation of a bite followed by two or more of: increasing pain during the first hour, radiating, regional, or generalised pain, and local or regional diaphoresis. Local envenoming was pre-defined as severe local pain, for which the patient was requesting analgesia, or that was preventing sleep while systemic envenoming was defined as the presence of three or more of the following: nausea, vomiting, headache, lethargy, malaise, and abdominal pain. Exclusion criteria were; patients younger than 8-years (because of the unreliability of the Verbal Numerical Rating Scale for assessment of pain in this group), prior administration of antivenom and presentation to hospital more than 36-h after the bite. All eligible patients were randomised to blinded treatment with RBSAV or placebo, according to a computer-generated randomisation code. Prior to the study intervention *all* patients received standardised analgesia. Each treatment kit contained 2-blinded vials of either RBSAV or normal saline solution (placebo). The content of each treatment pack was unknown to the treating clinician, patient and study investigators. The trial drug was administered (two vials of RBSAV or two vials of placebo) mixed in 200 mL of normal saline given over 20-min. The primary outcome was a clinically significant reduction in pain two hours after commencement of the study treatment. The Verbal Numerical Rating Scale (VNRS) was used as in the RAVE I study. A second primary outcome was number of subjects with a resolution of systemic features of envenoming within two hours in the subgroup with systemic envenoming.

Secondary outcomes were predefined as clinically significant reduction in pain and resolution of systemic features (if present) at four hours, administration of opioid analgesics (oral or parenteral) or further doses of antivenom after two hours, a clinically significant reduction in pain at 24 h, use of opioid analgesia after discharge, representation for medical care, acute systemic hypersensitivity reactions, and serum sickness defined as three or more characteristic symptoms (fever, malaise, rash, itchiness, myalgia, and arthralgia). Predefined subgroup analyses were planned for patients with systemic envenoming. A number of processes were put in place to ensure continued blinding of investigators to treatment groups until the final analysis was approved. Of the 224 patients, 112 were randomised to receive RBSAV and 112 were randomised to receive placebo. Twenty-six of 112 patients (23%) from the placebo arm had a clinically significant improvement in pain (treatment success) compared to 38 of 112 (34%) from the antivenom arm, Figure 1 (difference in favour of antivenom 10.7%; 95%CI: −1.1% to +22.6%; *p* = 0.10, not statistically significant). Systemic effects resolved after 2 h in nine of 41 patients (22%) in the placebo arm and nine of 35 (26%) in the antivenom arm. This was also not statistically significantly different. There was no significant difference in any secondary outcome between antivenom and placebo, including pain at four and 24 h, systemic features at four hours, and the use of rescue opioid analgesia and antivenom.

The authors acknowledged that the sample size may have been too small to completely exclude a small benefit from antivenom, however, the calculated number needed to treat (NNT) for benefit from antivenom was 10 patients. That is, for every 10 patients that receive antivenom only one patient will obtain significant pain relief. This is a very poor result for an analgesic treatment. Meta-analyses of effective analgesic treatments generally report a NNT of 2 to 4. Another limitation was that some cases were included according to a clinical diagnosis of latrodectism rather than a witnessed bite. This is a limitation of most latrodectism studies that might bias towards observing no treatment effect. Notably, a reanalysis of the primary outcomes including only cases in which the spider was identified resulted in the same outcomes. Finally, the authors noted that the measured pain outcomes had not been previously validated in this condition, which could result in either an under or over-estimation of the measured treatment effects. However, this would have affected both arms of the study, a similar primary outcome was used in two previous studies with one resulting in a negative outcome and the other resulting in a positive result [4,55]. The VNRS and VAS measures of pain have been found to be strongly correlated in both clinical research and in the Emergency Department for acute pain [56,57,58]. The VNRS has the added advantage of being easier to administer by the clinician and easier to understand by the patient.

The most recent multicentre, placebo-controlled randomised clinical trial of antivenom for latrodectism is by Dart et al. (2016) [52]. The authors published a *conference abstract* investigating the effectiveness and safety of the Analatro^®^ F(ab’)2 black widow antivenom. Sixty patients were randomised to one of two treatment groups, F(ab’)2 AV or saline placebo. Patients with moderate to severe pain measured using the VAS were enrolled and treated. Pain intensity was measured at baseline and every 30 min thereafter up to 150 min. Patients with moderate to severe pain or those who failed to achieve a clinically significant reduction in pain after the first dose received a second dose. The primary outcome measure was treatment failure, defined as failure to achieve and maintain a clinically significant reduction in pain for 48 h post-treatment. This primary outcome measure was reported to be “statistically significant” when analysed using a 1-sided Chi-squared test (Treatment Failures: 15/29 AV vs. 24/31 placebo). When the data is re-analysed using an appropriate 2-sided or 2-tailed Fisher’s exact test a non-significant p-value of 0.0576 is computed. The treatment failure rate is also just over 50% in the AV group. Treatment Success: 14/29 AV vs. 7/31 placebo is presented in Figure 1. As this RCT is a published abstract there is currently insufficient detail on the methods and other outcomes such as pain intensity for the two treatments over time and the frequency and types of adverse effects.

### 5.3. Meta-Analysis of Randomised Controlled Trials of Antivenom for Latrodectism

**Figure 2:**
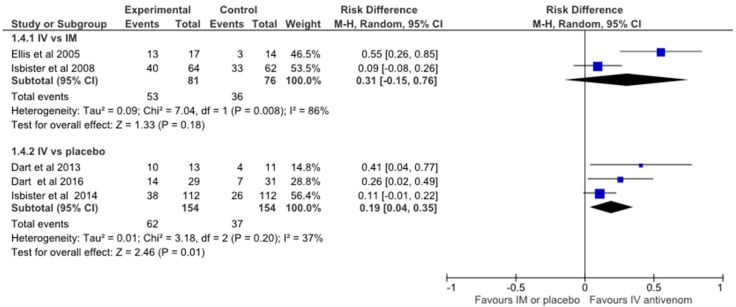
RevMan meta-analysis and Forest Plot of the five randomised controlled trials on antivenom for pain from latrodectism.

The forest plot (Figure 2) shows a small overall benefit of IV antivenom over IM antivenom or placebo on pain response. However, on closer inspection it is the smaller underpowered studies that tend to favour IV antivenom rather than the larger studies. If the risk difference for each study is converted to the number needed to treat in order to have a successful treatment outcome with IV antivenom, the smallest studies of Ellis et al. (2005), which had a sample size (SS) of 31, and Dart et al. (2013), which had a sample size of 24 have the lowest NNT of 2. When the sample size is larger the NNT is seen to increase to 4 for Dart et al. (2016), SS of 60 and to NNT = 9 and, 11 for Isbister et al. (2014) and (2008) (SS = 224 and SS = 126), respectively. The smaller studies show extreme differences that are not replicated in the larger studies. This is likely due to significant heterogeneity, as indicated in the RevMan analysis of heterogeneity suggesting non-random effects in the differences seen; bias such as selective reporting or statistical analysis. That the two trials with the largest effect sizes are industry sponsored raises concerns about whether this influenced the conduct or analysis of the trial. For example, the original sample size for Dart 2016 was 50, but was subsequently revised to 60. The trial is on the cusp of statistical significance, (falling each side, depending on choice of test), and it is unclear if there were interim or serial analyses with post hoc adjustment of sample size to achieve even this result. Irrespective of these potential biases, the pooled analyses which include these small studies results in a NNT of 5. If regarded as simply an analgesic therapy, this is a very expensive intervention of an uncertain benefit for four out of five patients. Further the adverse effect profile would be considered poor compared to most other analgesic alternatives.

## 6. Discussion

Latrodectism or envenomation by widow spiders is common worldwide and is the most common clinically significant spider envenomation. The venom contains alpha-latrotoxin, a neurotoxin that can result in an incapacitating syndrome of severe local, regional or systemic pain and autonomic features that, if left untreated, can last from several hours to days. Severe and persistent pain occurs in a half to two-thirds of *Latrodectus* cases and is the primary target of effective treatments. The literature on the clinical effectiveness of past and current treatments for latrodectism shows several phases as various treatments have swung in and out of favour. Earlier latrodectism drug treatments included calcium gluconate, muscle relaxants and opioid analgesia either combined or in isolation. These earlier treatments were based on anecdotal case reports and small case series with varying reports on patient effectiveness. A larger case series by Clark et al. in 1992 cast considerable doubt on the effectiveness of first-line calcium treatment for latrodectism. Calcium gluconate was then judged to be ineffective in symptom relief and opioid therapy in combination with muscle relaxants became the recommended treatment for latrodectism. Antivenom was primarily reserved for severe envenomation cases due to the perceived significant chance of hypersensitivity and serum sickness reactions. Further more rigorous studies suggest these risks were overstated, but also call into question whether antivenom has enough clinical benefits to warrant use at all.

An equine-derived IgG-F(ab)_2_ red-back spider antivenom (RBSAV) has been available for use in Australia for the last sixty years. It has a very low acute reaction rate and half of the allergic reactions following its use have been attributed to administration of undiluted antivenom by the intravenous route. The American equine derived whole IgG black widow spider *L. mactans* antivenom has also been available for the last sixty or so years. It is administered intravenously and has been regarded as having more frequent adverse effects than Australian RBSAV. The 2001 review by Clark suggests that the black widow spider antivenom has a similar safety profile to the red-back spider antivenom. Despite this only 3.8% of patients with clinical effects attributed to Latrodectism received black widow spider antivenom from 2000 to 2008 primarily due to the fear of adverse reactions to the antivenom.

The effectiveness of antivenom for red-back spider envenoming was considered excellent for many years based on case series collated by the manufacturer [37].The limitations and biases of these early reports of effectiveness are obvious in hindsight, but were only drawn to notice when better designed prospective studies noted low treatment effectiveness. This was followed by randomised comparator trials Ellis et al. (2005) and Isbister et al. (2008) which found no significant difference between intramuscular and intravenous RBSAV treatment on pain relief. Subsequent, multicentre placebo-controlled randomised trials also show poor treatment responses and no statistically significant differences. In all these studies there has been a non-significant trend towards a minor positive effect. However, the clinical responses in these prospective studies have consistently been much less than the older uncontrolled studies reported. Further, the differences between AV and control groups have generally been much less than the decline due to other treatments and the natural history of resolution.

In conclusion, this review suggests that current and past treatments are generally ineffective in providing rapid adequate symptom relief for patients with *Latrodectus* spp. envenoming. Ongoing investigation into the mechanism of latrodectism is essential as this may lead to more effective treatments being developed. Investigation into alternate pain treatments utilising placebo-controlled randomised clinical trials are urgently needed.

## 7. Materials and Methods

We searched MEDLINE, EMBASE and Google Scholar from 1946 to December 2016 and included any clinical studies of latrodectus envenoming with treatment, including antivenom and other medical treatments such as analgesia and calcium. The following keywords were used: “red-back spider”, “widow spider”, “latrodectism”, “antivenom” and “treatment”. Reference lists of identified articles were searched to find additional publications. Only articles less than 40 years old and in English were reviewed. We identified a total of 48 studies of which 34 were included for review. There were two full-publication and one abstract placebo-controlled randomised trials, two randomised (no placebo) comparative trials, 14 case series (including two abstracts), 14 case reports, and one letter discussing the effectiveness of treatments for latrodectism. Four of the case reports and two of the case series were on paediatric patients. We performed a meta-analysis on the five randomised controlled trials that investigated the effectiveness of antivenom on pain from latrodectism using RevMan 5 software [Review Manager (RevMan) Version 5.3. Copenhagen: The Nordic Cochrane Centre, the Cochrane Collaboration, 2014]. We compared the Ellis et al. (2005) and Isbister et al. (2008) RCT’s of IV Antivenom versus IM Antivenom (both using RBSAV, CSL Ltd. Melbourne, Australia). We then compared the Dart et al. (2013), Dart et al. (2016) (*Latrodectus* (Black Widow) F(ab)2 (Analatro, Instituto Bioclon S.A. de C.V., Mexico City, Mexico) and Isbister et al. (2014) (RBSAV, CSL Ltd., Melbourne, Australia) RCT’s of IV Antivenom versus saline placebo. We calculated the risk differences and 95% CI using the Mantel-Haenszel method, and a random effects model due to the different outcome definitions for the pooled estimate. We presented the outcomes in a Forest Plot again using RevMan V5.3 and calculated the Numbers Needed to Treat (NNT) for each study.

## Figures and Tables

**Table 1:** Summary of non-randomised clinical studies for latrodectism using earlier/alternate treatments.

Study Author/s	Type of Study	N	Study Arms	Efficacy Outcome	Design Problems	Study Conclusion/s
Key, 1981 [16]	Prospective observational study, with cross over to the alternative treatment with treatment failure	13/10	calcium gluconate vs. methocarbamol	Not specified	Small study	Calcium gluconate should remain the treatment of first choice for latrodectism as it had a better outcome than methocarbamol
Ryan, 1984 [17]	Retrospective, case series	6/2	IV and oral dantrolene sodium vs oral dantrolene sodium	Not specified	Small uncontrolled study	Medication side effects noted as mild. Further studies on optimal dose and efficacy compared to other treatments necessary
Timms & Gibbons, 1986 [18]	Retrospective, case series	11	9 pts received IV calcium gluconate with diazepam or methocarbamol, 1 received methocarbamol, meperidine hydrochloride and nasogastric suction and 1 received calcium gluconate and methylprednisolone sodium succinate. Two of the 11 patients also received L. mactans antivenom 2.5 mg IV. 7/11 pts required narcotic analgesics for pain.	Recovery Time	Small, uncontrolled non-comparative study	Latrodectism is responsive to calcium gluconate with muscle relaxant. AV is effective but rarely necessary and may result in serum sickness

**Table 2:** Summary of non-randomised clinical studies for treatment of latrodectism with antivenom.

Study Author/s	Type of Study	N	Treatment	Efficacy Outcome	Design Problems	Study Conclusion/s
Sutherland & Trinca, 1978 [37]	Retrospective case series	2073/44 (IM/IV)	RBSAV IM/IV	Not specified	Reporting bias e. Data from questionnaires included with AV therefore no data on RBS bites that did not require AV, missing data. Multiple outcomes.	Difficult to elucidate—Signs and symptoms of envenomation and general perception of ‘good’ outcome after antivenom but no quantitation of outcome data presented.
Jelinek et al., 1989, [38]	Retrospective case series	150	RBSAV by IM if systemic symptoms present, *n* = 32.	Not specified	ED presentations only—May have selected more serious cases.	11/32 pts given AV by IM needed more than 1 ampoule. Authors suggest this is because WA red-back spider is more venomous.
Clark, 1992, [1]	Retrospective case series	163	IV opioids and benzodiazepines +58 patients received L mactans AV by IV	Pain relief ≥30 min after treatment administered.	Retrospective review of case notes—There was an attempt to standardise with operational definitions for data entry; strict inclusion/exclusion criteria; small group of toxicologists looking after patients. Retrospective telephone follow up for delayed AV reactions could only be done in 9 patients.	Calcium gluconate was found to be ineffective while L mactans AV significantly shortened the duration of symptoms in severe envenomations.
Mead & Jelinek, 1993, [39]	Retrospective *Paediatric* case series	241	21% of children received RBSAV	Not specified	Poorly defined criteria for systemic envenomation—May result in under-reporting.	Definite bite by RBS defined; syndrome in children primarily similar to adults. Use of AV comparable to previous studies in older age groups; however, no child received more than one ampoule. Results suggested that contrary to current opinion at the time [40,41,42,43] children may not be at an increased risk of morbidity from latrodectism.
Mollison et al., 1994, [44]	Retrospective ICU case series	32	26 patients received RBSAV	Partial, features of red-back spider envenomation in Australian Aborigines determined by collecting data on standard forms	Only severe cases (ICU)-reported, no data kept on patients seen in ED.	Annual incidence of severe RBS bite calculated. Unlike earlier series this study had a predominance of female patients.
Dzelalirja & Medic, 2003, [45]	Retrospective case series & lab analysis	32	21 patients received European WSAV	Pain intensity and duration after antivenom	Inadequate power for AV vs no AV comparison	Latrodectism in Northern Dalmatia presents with severe clinical symptoms. AV is advisable in the treatment of all afflicted patients.
Trethewy et al., 2003, [46]	Retrospective *Paediatric* case series	54	45 patients received IM RBSAV	Not specified	Qualitative not quantitative assessment of symptoms-patient notes reviewed retrospectively. Clinical signs/symptoms difficult to define in children. For example, only 50% patients had a bite site identified.	No statistically significant difference between age groups for local or systemic envenomation even though the older children were twice the weight of younger group. This was in contrast to previous studies suggesting envenomation is different in children [37,47]. 46 children classified as systemic envenomation and 38 of these received RBSAV.
Isbister & Gray, 2003, [11]	Prospective case series on calls to NSW, QLD & WA Poison Information Centres and presentations to two Hospital ED (Sydney & Darwin).	68 with definite RBS bite	6 patients received IM RBSAV	Reported proportion of 6 patients with no pain at 24 h.	Study lacked power to compare IM AV and no treatment.	The severity of envenomation should be defined by the severity of pain and systemic features, AND also to the duration of these effects. Only 1/6 of patients receiving AV were pain-free at 24 h, an unacceptable treatment effect. IM RBSAV was no better than no treatment when all patients were followed up over a week.
Nordt et al., 2010, [48] ^	Retrospective case series	96	All patients received *L. mactans* AV	Not specified	Published abstract only therefore limited methodology explained.	Symptomatic treatment for black widow spider envenomation includes opioid pain control and muscle relaxants. Definitive treatment includes AV to neutralize venom. Noted that adequate pain control is often difficult to achieve. Although derived from horse serum, hypersensitivity reactions appear to be mild and rare but further prospective studies are required to confirm.
Monte et al., 2011, [7]	Retrospective case series on *Latrodectus* spp. exposures & treatment reported to US National Poison Data System over 8 years.	9872	Patients received benzodiazepines, calcium, IV fluids or dilution/wash of bite site. 374 (3.8%) patients received *L. mactans* AV.	Not specified	Only cases reported to US poison centers reviewed. Dataset contained no information on dosing or timing of treatments. Unable to differentiate adverse drug reactions for the different treatments as most patients received multiple therapies and the dataset does not attribute ADRs to the specific treatment given.	Few patients received AV although it was associated with shorter symptom duration (<24 h) in moderate and major severity groups. There was no evidence of shorter symptom duration in patients who received benzodiazepines or calcium. Adverse drug reactions were more common in patients receiving benzos and AV.
Basanou et al., 2015, [49] ^	Retrospective case series of Poisons Centre consults	53	Mod to severe cases (23/53) treated with IV benzos, *n* = 6, IV opioids *n* = 5 or combination of both *n* = 12. IV *L. mactans* AV in 5 patients with severe systemic effects.	Not specified possibly “symptomatic control”	Published abstract only therefore limited methodology explained.	Opioid analgesics combined with muscle relaxants, such as benzodiazepines, are generally effective at symptomatic control. In selected severe cases antivenom is the most efficacious therapy available.

^ Conference publication only available. RBSAV: Red-back spider antivenom.

**Table 3:** Summary of randomised controlled clinical trials of antivenom treatment for latrodectism.

Study Author/s	Number in Each Arm	Blinded	Allocation Concealed	AV Dose/Brand	Primary Outcome/s	Conclusion/s
Ellis et al., 2005 [27]	15/18 (IM AV/IV AV)	Yes	Yes (1 from each arm was unblinded and removed from the analysis)	500 units RBS Antivenom (CSL Ltd., Melbourne, Australia)	Partially defined 2: number of AV ampoules used and pain scores up to 2 h.	Both IM and IV RBSAV were deemed effective as after 1 h-pain VAS fell, with a better outcome at 24 h for the IV route. There were some major and acknowledged study limitations including too small sample size, and an insensitive primary outcome (N of AV ampoules) Thus the study was underpowered and inconclusively negative.
Isbister et al., 2008 [4]	62/64 (IM AV/IV AV)	Yes	Yes	1 or 2 vials of RBS Antivenom (equine Fab’2 500 U/vial, CSL Ltd., Australia)	Pain at 2 h after treatment administered.	No real difference in IV and IM routes. Further, RBSAV may provide no benefit over placebo.
Dart et al., 2013 [53]	13/11 (AV/placebo)	Yes	Yes	3 vials of Antivenom *Latrodectus* (Black Widow) Equine Immune F(ab)2 (Analatro^®^, Instituto Bioclon S.A. de C.V., Mexico).	VAS pain intensity at 150 min after treatment administered.	No difference between BWAV and placebo pain VAS *
Isbister et al., 2014 [54]	112/112 (AV/placebo)	Yes	Yes	2 vials of RBS Antivenom (equine Fab’2 500 U/vial, CSL Ltd., Australia)	Yes, pain at 2 h after treatment administered.	No difference between RBSAV and placebo pain VNRS **
Dart et al., 2016 [52] ^	AV/placebo	Yes ^	Yes ^	1 to 2 doses of F(ab’)2 Antivenom (Analatro^®^)	Treatment failure-pain at 48 h after treatment administered.	F(ab’)2 AV was effective at reducing moderate to severe pain caused by latrodectism, however a 1-sided hypothesis test was used. No serious safety concerns were identified ^

* VAS: Visual Analogue Scale; ** VNRS: Verbal Numerical Rating Scale. ^ Published conference abstract only therefore limited information on methodology and outcomes available for review.
